# Assessing the impact of climate change on the potential distribution of *Keteleeria evelyniana* Mast. in southwest China: a Maxent modeling approach

**DOI:** 10.3389/fpls.2025.1561031

**Published:** 2025-04-25

**Authors:** Yuan Feng, Guanghui Dai, Hua Li, Yong Chai, Rui Bao, Meng Wang, Chunlin Luo, Yangping Qin

**Affiliations:** ^1^ Yunnan Academy of Forestry and Grassland, Kunming, Yunnan, China; ^2^ Yunnan Key Laboratory of Biodiversity of Gaoligong Mountain, Kunming, Yunnan, China; ^3^ Gaoligong Mountain Forest Ecosystem Observation and Research Station of Yunnan Province, Kunming, Yunnan, China; ^4^ Southwest Survey and Planning Institute, National Forestry and Grassland Administration, Kunming, Yunnan, China; ^5^ State Key Laboratory of Efficient Production of Forest Resources, Institute of Forest Resource Information Techniques, Chinese Academy of Forestry, Beijing, China

**Keywords:** *Keteleeria evelyniana* Mast., Maxent model, climate change, potential distribution, environmental factor

## Abstract

*Keteleeria evelyniana Mast.*, which is widespread in southwestern China, is valuable for studying under different future climate scenarios to assess potential distribution shifts in response to climate warming. Understanding these changes can provide theoretical support for species conservation, rational utilization, ecological restoration, and management of *K. evelyniana* habitats. The Maxent model was optimized using the package of ENMeval to adjust the Regularization Multiplier (RM) and Feature Class Combinations (FC) parameters. Utilizing 221 effective distribution points and 33 environmental variables, the potential distribution of *K. evelyniana* in current and future climate scenarios was predicted, with the key environmental variables analyzed. The model with FC = LQ and RM = 0.5, demonstrated low complexity, minimal overfitting, and high accuracy, achieving an AUC value of 0.946 with a standard deviation of 0.011. Under the current climate conditions, 68% of the suitable areas for *K. evelyniana* were focused on Yunnan Province, with additional areas in western and southwestern Guizhou, southwestern Sichuan, and the southeastern Xizang Autonomous Region. In various future climate scenarios, the suitable areas for *K. evelyniana* gradually decreased, with a maximum reduction of 33%. Simultaneously, the centroids of these areas are expected to migrate northward by up to 33 km. Temperature was the dominant factor affecting its distribution (77.8%), whereas the effects of soil variables and altitude were significant. This study clarified the current distribution of *K. evelyniana*, projected the potential shifts under different future climate scenarios, and identified the main environmental factors affecting the distribution. These findings offer valuable theoretical support for the conservation, ecological restoration, and sustainable use of *K. evelyniana*.

## 1 Introduction

Species distribution is closely related to climate, with climate change serving as the primary driver for shifts in these distributions (Paz-Kagan et al., 2021; Lawlor et al., 2024). Climate change not only alters the geographical distribution of species but also significantly affects their abundance, diversity, composition, and ecosystem functioning (Bellard et al., 2012; Dieleman et al., 2015; Feeley et al., 2020; Ruiz-Labourdette et al., 2012). According to the Intergovernmental Panel on Climate Change (IPCC) AR6 Synthesis Report (IPCC, 2023), global warming is attributed to greenhouse gas emissions caused by human activities. From 2011 to 2020, the global average surface temperature was 1.1°C higher than that from 1850 to 1900 and is projected to surpass the 1.5°C threshold by 2021–2040 (Calvin et al., 2023). This trend indicates significant shifts in the suitable ranges of species, with warming likely to drive species toward higher altitudes or latitudes (Wilson et al., 2007; Lawlor et al., 2024), whereas some may face extinction risks (Thomas et al., 2004). Therefore, in-depth study of species distribution patterns related to climate change is crucial for developing scientific strategies for species conservation and germplasm resource management. Such research not only supports biodiversity conservation but also provides a scientific foundation for activities such as species introduction and cultivation.

The Species Distribution Model (SDM), also known as an Ecological Niche Model (ENM), is a mathematical tool adopted to predict the geographic distribution of species based on data on species presence or abundance and environmental factors (Elith and Leathwick, 2009). When combined with global climate models, SDMs can project species range changes in future climate scenarios. Common SDMs methods include Random Forest, Maximum Entropy, eXtreme Gradient Boosting Training, and more than ten others (Thuiller et al., 2012). Among these, Maximum Entropy has been widely applied in areas such as assessing climate change impacts on species distribution (Soilhi et al., 2022; He et al., 2023; Shi et al., 2024), protecting endangered species (Gao et al., 2022; Zhao et al., 2022; Deng et al., 2024), prevention and control of invasive species (Low et al., 2021; Sorbe et al., 2023), pest management (Kuprin et al., 2024; Wei et al., 2024), and geological disaster prediction (Boussouf et al., 2023; Qasimi et al., 2024) because of its advantages such as high predictive accuracy, reliance solely on occurrence data, and robustness to sample size limitations (Pearson et al., 2007; Phillips and Dudık, 2008; Elith et al., 2011).

*K. evelyniana*, an evergreen tree in the Pinaceae family and genus *Keteleeria*, is a relict species from the Tertiary period (Du, 2022). It is a valuable timber and reforestation species in the southern plateau region, with significant economic importance and a crucial role in ecological conservation (Du, 2022). According to the Plants of the World Online website, *K. evelyniana* is distributed throughout East Asia, including China, Laos and Vietnam (https://powo.science.kew.org/). Within China it grows mainly in Yunnan Province, western and southwestern Guizhou, and from the Anning River Basin to the western Dadu River Basin in Sichuan Province (Editorial Committee of flora of China, Chinese Academy of Sciences, 2004). Listed as Near Threatened (NT) on the IUCN Red List, existing research has primarily focused on its chemical composition (Fu et al., 2008; He et al., 2011), silviculture (Wang et al., 2022; Zhou et al., 2023b), plant-fungal symbioses (Ge et al., 2012) and community structure (Li et al., 2013; Tang et al., 2024). However, there is still a knowledge gap regarding how its distribution can vary under future climate scenarios and which environmental factors predominantly influence its range. Based on the environmental variables and distribution data of *K. evelyniana*, this study aimed to (1) predict its potential distribution in southwest China, (2) analyze the dominant environmental variables influencing its distribution, and (3) explore how its suitable habitats may change under different future climate scenarios. This study provides a theoretical foundation for species conservation, rational resource use, and ecological restoration of *K. evelyniana*.

## 2 Materials and methods

### 2.1 Data sources and processing

#### 2.1.1 Species occurrence data

**Figure 1:**
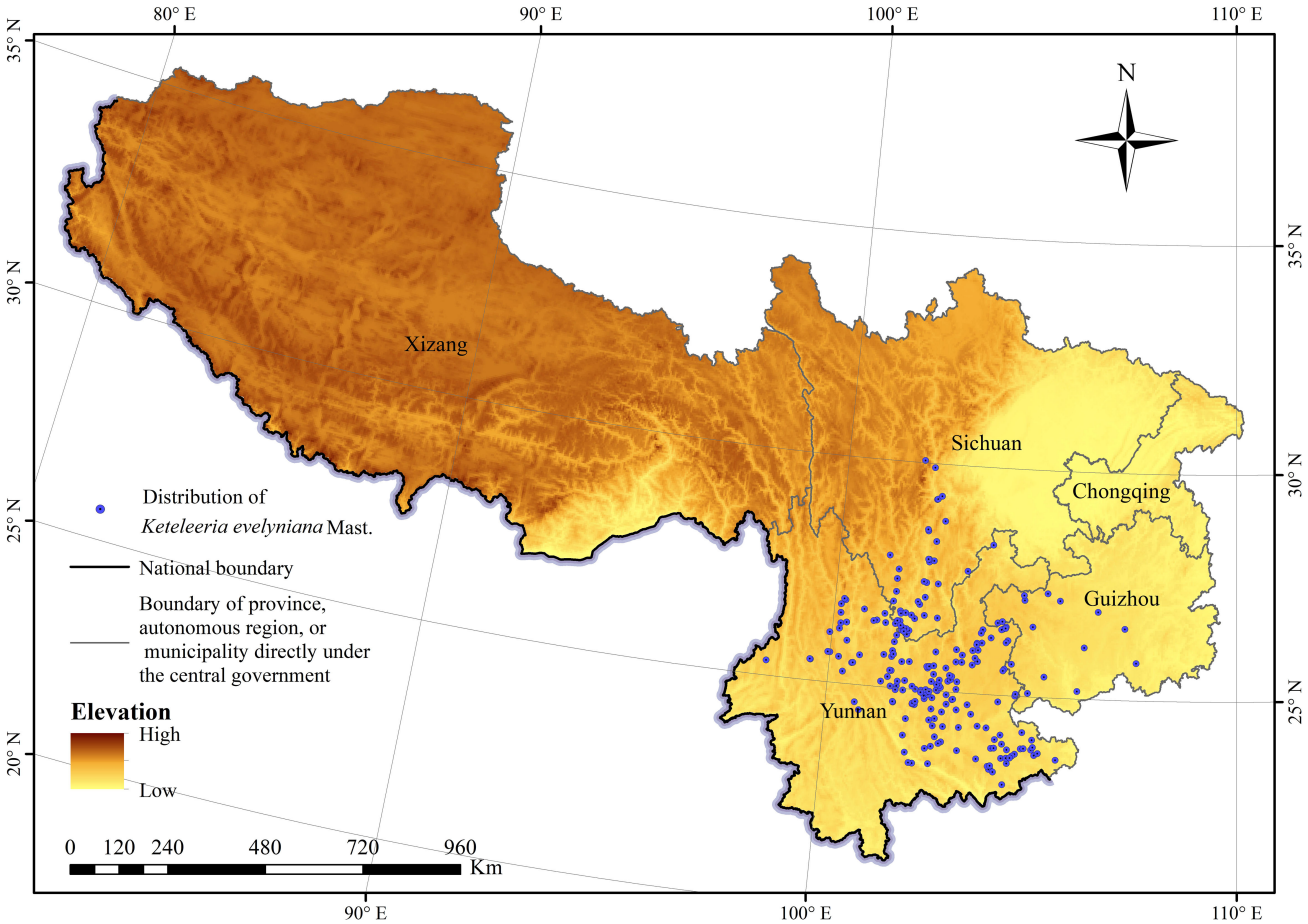
Distribution of *Keteleeria evelyniana* Mast. in southwest China.

The main sources of distribution data for *K. evelyniana* were field surveys, Global Biodiversity Information Facility (GBIF, https://www.gbif.org/), Chinese Virtual Herbarium (CVH, https://www.cvh.ac.cn/), and literature (Tang et al., 2017; Du, 2022). Duplicate and invalid distribution points were removed, and to reduce spatial autocorrelation and prevent overfitting, a grid cell size of 2.5′×2.5′ latitude and longitude (consistent with the spatial resolution of environmental variables) was used with one record per cell (Yan et al., 2021). A total of 221 geographical occurrences of *K. evelyniana* were included in further analysis (**Figure 1**), 85 from field surveys, 77 from GBIF, 3 from CVH and 56 from literature.

#### 2.1.2 Environmental variables

To study the potential distribution of *K. evelyniana*, 33 environmental variables were used, including 19 bioclimatic, 3 topographic, and 11 soil variables, as detailed in **Appendix Table S1**. The bioclimatic variables included both current data and future climate scenario projections for the following periods: the current period and four subsequent 20-year intervals from 2021 to 2100. Current bioclimatic data were extracted from WorldClim 2.1, averaged over 1970–2000 (Fick and Hijmans, 2017), whereas future bioclimatic data were obtained using the Beijing Climate Center Climate System Model 2 Medium Resolution (BCC-CSM2-MR) under the Coupled Model Intercomparison Project Phase 6 (CMIP6). The scenarios of SSP5-8.5, SSP2-4.5, and SSP1-2.6 were selected to project the future distribution of *K. evelyniana*, representing the conventional, moderate, and sustainable development pathways with radiative forcings of 8.5, 4.5, and 2.6 W/m^2^, respectively, by 2100 (He et al., 2023; Shi et al., 2023). Each scenario involved two time periods: 2021–2040 (2030s) and 2061–2080 (2070s). The elevation data were sourced from WorldClim 2.1, whereas the aspect and slope were derived from the spatial analysis using GIS software. Soil data were obtained from the SoilGrids 250 m system, with each soil variable (except Ocs) divided into six profile depths (Poggio et al., 2021), weighted, and averaged by depth to obtain the final values. The spatial resolution of all environmental variables was 2.5 arc minutes.

Environmental variables often exhibit varying degrees of collinearity. To prevent model overfitting and enhance simulation accuracy and practical usability, these variables should be screened before being used in the Maxent model (Miller, 2010). Variables that exhibited a correlation of less than 0.8 (**Appendix Figure A1**), and an initial contribution value greater than 1% to Maxent’s prediction was maintained (**Appendix Table S1**). Finally, six environmental variables were adopted, as listed in **Table 1**.

**Table 1:** Contribution of the six environmental variables to the *K. evelyniana* Maxent prediction model.

Environment variables	Variable description	Unit	Percent contribution (%)	Permutation importance (%)	Regularization training gain only with this variable	Regularization training gain without this variable
Bio6	Min temperature of coldest month	°C	**39.3**	**45.8**	0.0557	**1.8310**
Bio4	Temperature seasonality (standard deviation ×100)	–	**38.5**	**31.2**	**1.2336**	**1.7856**
Sand	Proportion of sand particles (> 0.05 mm) in the fine earth fraction	g/100g (%)	**10.8**	2.0	**0.9840**	1.8360
Soc	Soil organic carbon content in the fine earth fraction	g/kg	8.2	9.0	**1.3736**	1.8426
Bdod	Bulk density of the fine earth fraction	kg/dm³	1.8	0.7	0.7600	1.8393
Elev	Elevation	m	1.5	**11.3**	0.2238	**1.7885**

Bolding indicates the top three contributing variables.

Administrative division base maps in this study were sourced from the Standard Map Service Platform (http://bzdt.ch.mnr.gov.cn/index.html) with the map approval number GS (2016) 2923. The base maps were used without modification.

### 2.2 Maxent model optimization, construction, and evaluation

#### 2.2.1 Maxent model optimization

Default parameter settings for Maxent models are commonly used in species distribution modeling, but they can result in overly complex models that are prone to overfitting (Shi et al., 2024; Warren et al., 2014; Zhao et al., 2021b). Among the parameters influencing Maxent model outcomes, the Regularization Multiplier (RM) and Feature Class Combinations (FC) have the greatest impact. In this study, we tuned these parameters using the ENMeval package (Kass et al., 2021), setting the RM values from 4 to 0.5 in 0.5 decrements and the FC values to LQHPT, LQHP, LQH, H, LQ, and L, creating 48 parameter combinations, following previous studies (Shi et al., 2024; Zhao et al., 2021a). The model performance was evaluated using the 10% training omission rate (OR10), the difference between the training and test AUC (AUC.DIFF), and Akaike Information Criterion correction (AICc) (Pearson et al., 2007; Warren and Seifert, 2011). The AICc reflects the model’s complexity and goodness of fit, with the model having the smallest AICc value (delta.AICc = 0) considered optimal, and models with delta.AICc < 2 considered highly reliable. In addition, AUC.DIFF and OR10 were used to assess the degree of overfitting.

#### 2.2.2 Maxent model construction

The 221 occurrence records of *K. evelyniana* and six environmental variables were input into Maxent V 3.4.4 (https://biodiversityinformatics.amnh.org/open_source/maxent/) to predict its potential distribution across different time periods, with the cross-validation method repeated 10 times. RM and FC applied the previously optimized parameters, whereas the other settings adhered to Maxent’s default values. The model outputs (.asc) were converted into raster data using ArGIS software, and the potential distribution of *K. evelyniana* was categorized into four suitability groups using the Jenks natural breaks method (He et al., 2023; Shi et al., 2024): unsuitable area (0-0.11), low suitable area (0.11–35), medium suitable area (0.35–0.63), and high suitable area (0.63–1.00). Use the “Raster to Polygon” tool to convert the classified raster data to vector data, define the projection, and calculate the area of each suitability group. Additionally, the SDM toolbox tool was applied to analyze the centroid location and migration trends of suitable areas for *K. evelyniana* across different future time periods (Brown et al., 2017).

#### 2.2.3 Maxent model evaluation

The area under the Receiver Operating Characteristic curve (AUC), which ranged from 0 to 1, was used to evaluate the accuracy of the model (Elith et al., 2006). A higher AUC value indicates better predictive performance. An AUC value greater than 0.9 reflects the excellent predictive performance, a value between 0.7 and 0.9 indicates the good predictive ability, and an AUC from 0.5 to 0.7 signifies the average predictive ability (Swets, 1988). Equation 1 shows the calculation of AUC.


(1) $$AUC=\frac{1}{\left(a+c)(b+d\right)}\sum_{i=1}^{b+d}\sum_{j=1}^{a+c}\emptyset (X_{i},Y_{j})$$

Where, if Y > X, then $\emptyset (X_{i},Y_{i})$ = 1, if Y = X, then $\emptyset (X_{i},Y_{i})$ = 0.5, otherwise, $\emptyset (X_{i},Y_{i})$ = 0. X_i_ and Y_j_ are the predicted values for the unmeasured sample *i* and the measured sample *j*, respectively. Additionally, *a* denotes the true positive (TP), *b* the false positive (FP), *c* the false negative (FN), and *d* the true negative (TN).

A combination of percentage contribution, permutation importance, and jackknife tests was used to identify the dominant environmental factors.

## 3 Results

### 3.1 Model optimization and accuracy evaluation

**Figure 2:**
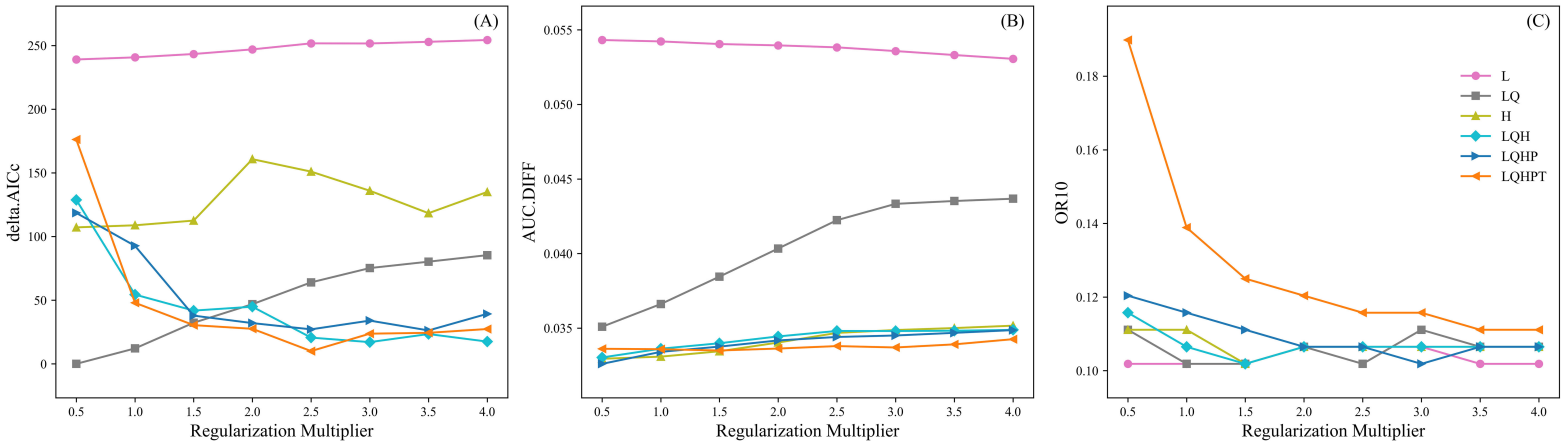
Changes in delta.AICc **(A)**, AUC.DIFF **(B)**, and OR10 **(C)** of the *K. evelyniana* Maxent model under different parameter combinations of FC and RM.

RM and FC were optimized in this study to reduce model complexity and improve fit. With RM set to 1 and FC set to LQHPT as the default, the model had a delta.AICc of 47.95. In contrast, the model with RM set to 0.5 and FC set to LQ had the smallest delta.AICc value of 0 (**Figure 2A**), indicating the lowest complexity. Further comparison showed that under FC=LQ and RM=0.5, the AUC.DIFF increased by 4.48% (**Figure 2B**), whereas OR10 decreased by 20.00% (**Figure 2C**) compared to the default settings. Therefore, FC=LQ and RM=0.5 were selected as the optimal settings.

**Figure 3:**
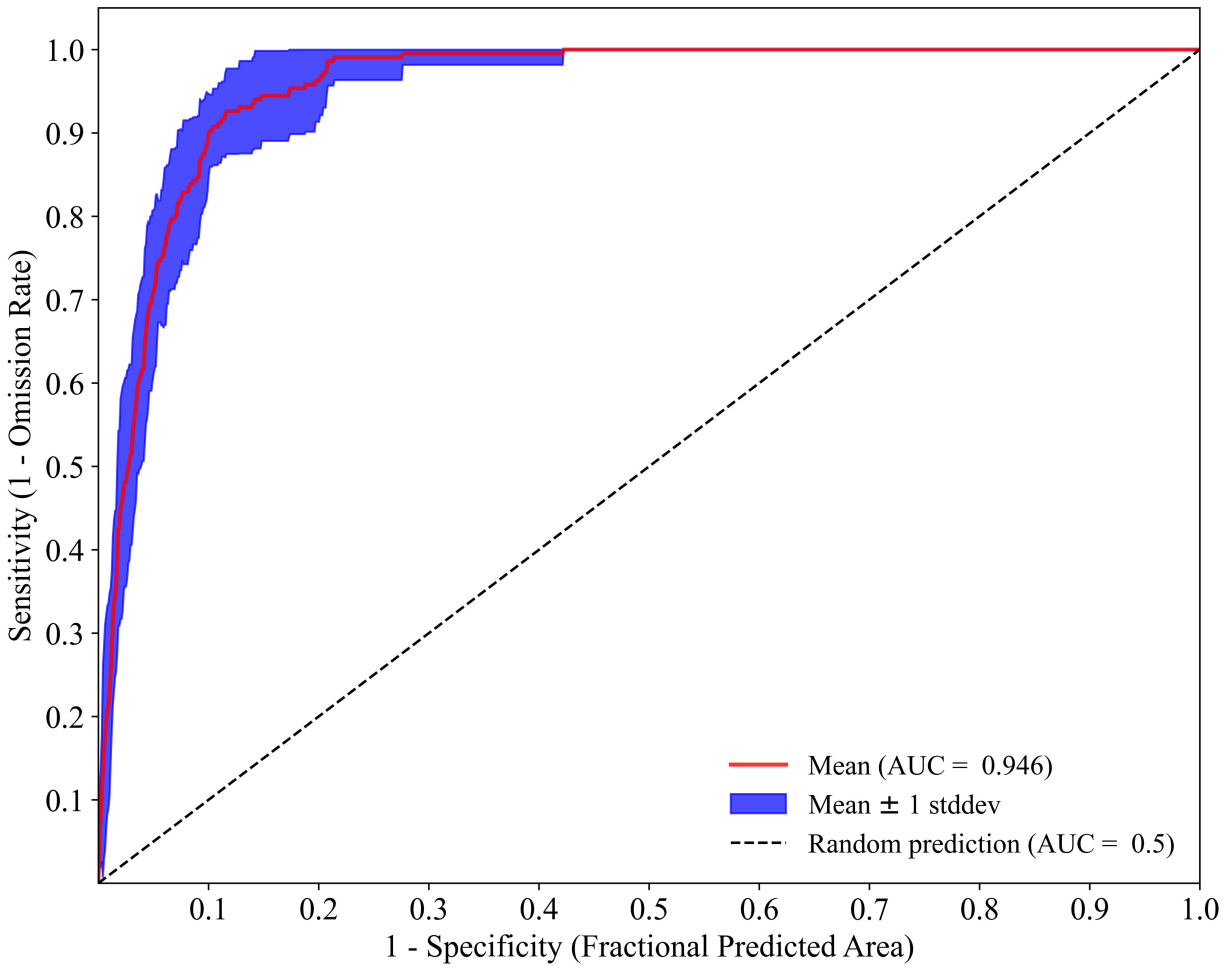
ROC analysis of Maxent model for predicting the distribution of *K. evelyniana*.

The ROC curve of the *K. evelyniana* Maxent model is shown in **Figure 3**, with an AUC value of 0.946 and a standard deviation of 0.011. This demonstrated that the model presented excellent predictive ability and accurately predicted of the potential distribution of *K. evelyniana*.

### 3.2 Dominant environmental variables and response curve analysis

The contributions of each environmental variable to the *K. evelyniana* Maxent model are presented in **Table 1**. The top three contributing variables were the lowest temperature of the coldest month (Bio6), temperature seasonality (Bio4), and the proportion of sand particles (> 0.05 mm) in the fine earth fraction (Sand), with a combined contribution of 88.6%. The highest permutation importance was attributed to Bio6, Bio4, and elevation (Elev), with a cumulative contribution of 88.3%. When considering individual variables for prediction, soil organic carbon (Soc), Bio4, and Sand were the most significant. Removing Bio4, Elev, and Bio6 from the model resulted in the greatest loss of regularization training gain. Overall, temperature variables (Bio4 and Bio6) were the most critical environmental factors influencing the distribution of *K. evelyniana*, contributing 77.8%, whereas soil variables and elevation also played significant roles.

**Figure 4:**
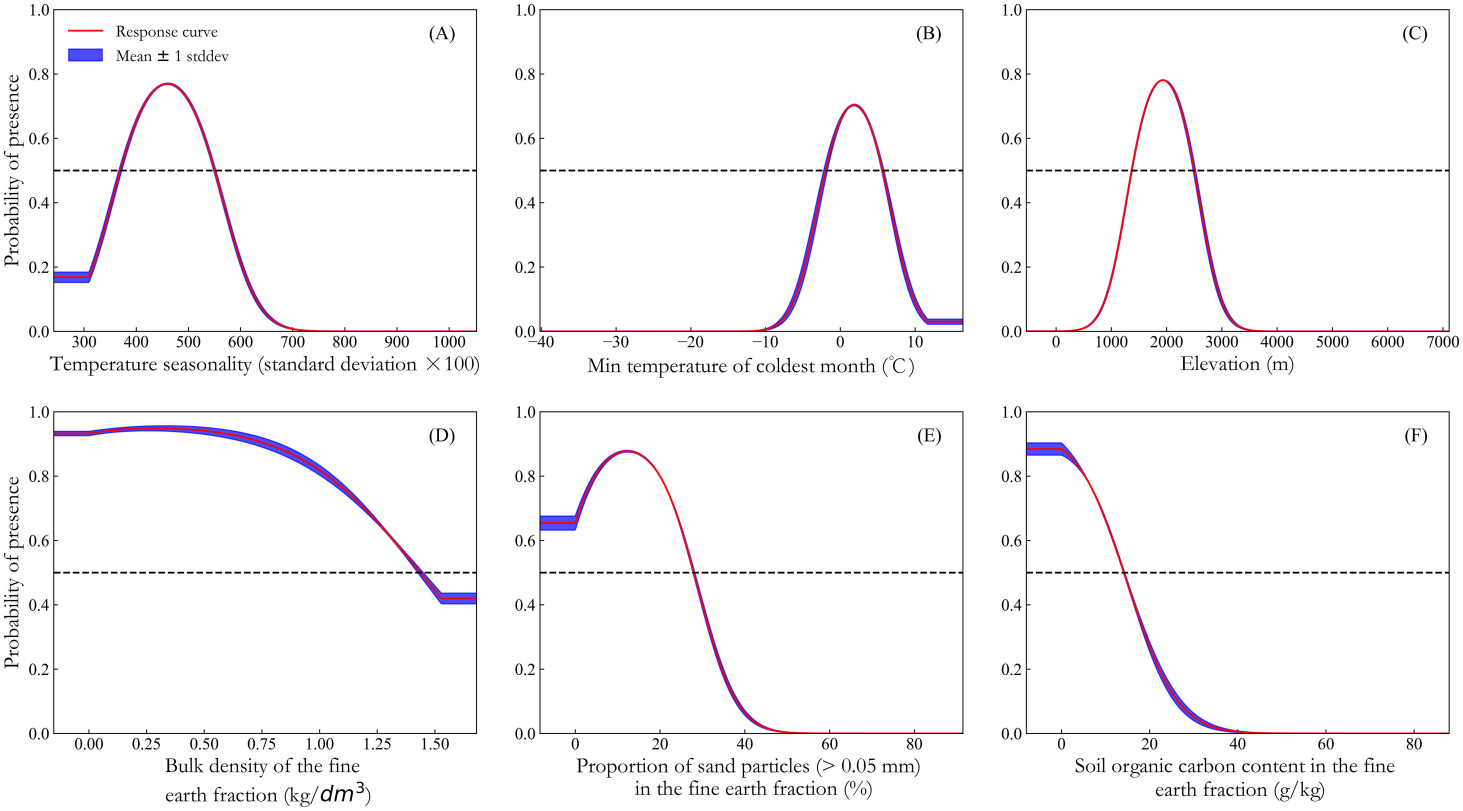
Response curves of dominant environmental variables: **(A)** temperature seasonality; **(B)** min temperature of coldest month; **(C)** elevation; **(D)** bulk density of the fine earth fraction; **(E)** proportion of sand particles (> 0.05 mm) in the fine earth fraction; **(F)** soil organic carbon content in the fine earth fraction.

**Figure 4** illustrates the response curves of the probability of *K. evelyniana* presence on the dominant environmental variables. According to previous studies (Shi et al., 2024; Zhao et al., 2021b), when the probability of presence exceeds 0.5, the corresponding environmental variable value can be considered favorable for plant cultivation. Based on this, suitable ranges for *K. evelyniana* growth were identified with Bio4 ranging from 370 to 550 (**Figure 4A**), Bio6 from -1.93 to 5.65 °C (**Figure 4B**), Elev from 1373.8 to 2490.5 m (**Figure 4C**), Bdod from 0 to 1.44 kg/dm³ (**Figure 4D**), Sand from 0 to 27.79% (**Figure 4E**), and Soc from 0 to 14.07 g/kg (**Figure 4F**).

### 3.3 Potential distribution and dynamic changes of *K. evelyniana*

#### 3.3.1 Potential distribution of *K. evelyniana* under current climate conditions

**Figure 5:**
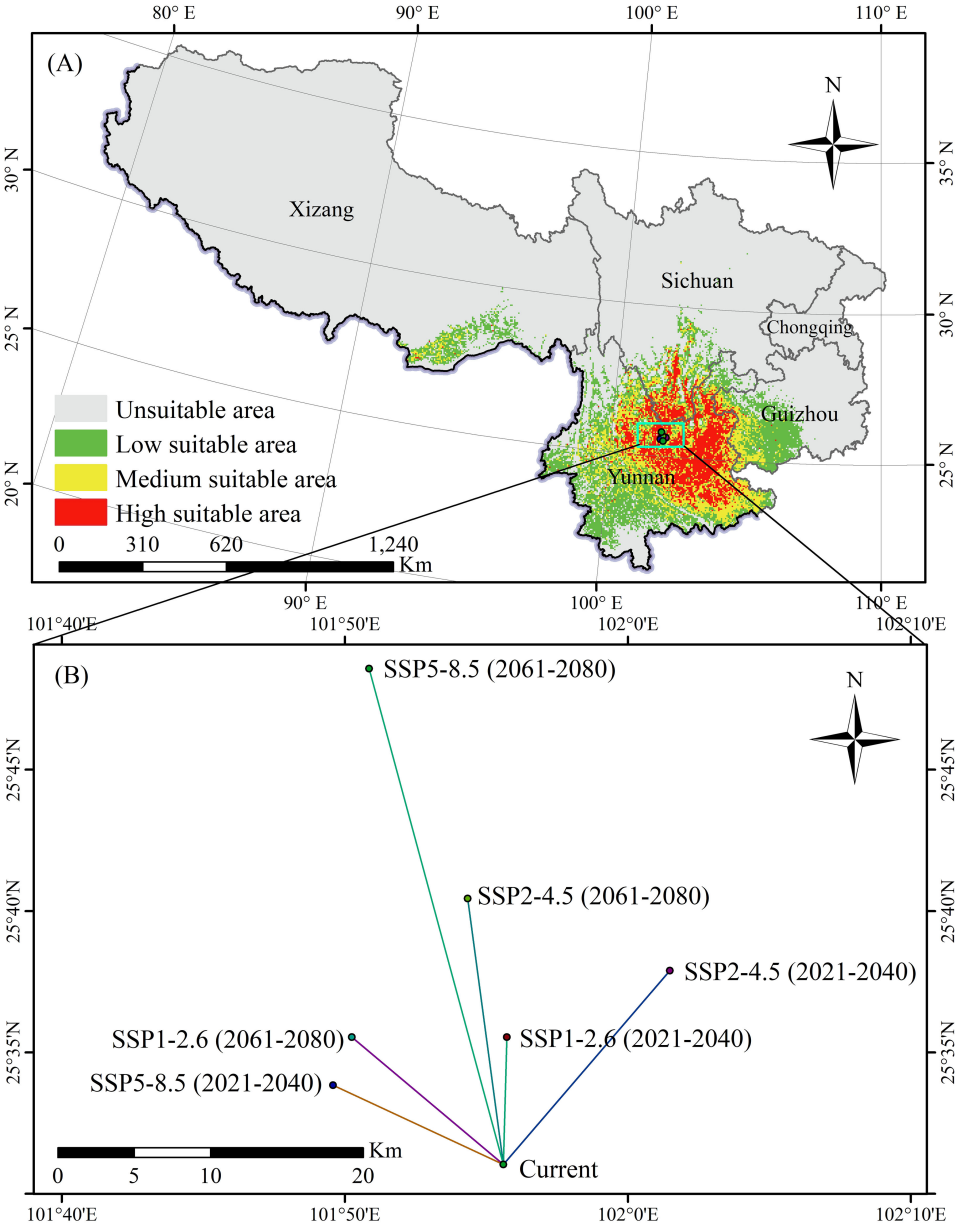
Potential distribution of *K. evelyniana* under current climate conditions **(A)** and centroid migration under future climate scenarios **(B)**.

The suitable areas for *K. evelyniana*, attributed to the Maxent model prediction under current climate conditions, are presented in **Table 2** and **Figure 5A**. The total suitable area was 40.94×10^4^ km^2^, with a 9.13×10^4^ km^2^ high suitable area, 12.01×10^4^ km^2^ medium suitable area, and 19.80×10^4^ km^2^ low suitable area. In southwest China, the potential distribution was primarily concentrated in Yunnan Province, which accounted for 68% of the suitable area. Others were located in western and southwestern Guizhou Province, southwestern Sichuan Province, and southeastern Xizang Autonomous Region, whereas no suitable areas were found in the Chongqing Municipality.

**Table 2:** Suitable area for *K. evelyniana* under current and different future climate scenarios.

Scenarios	Period	Total suitable area (×10^4^ km^2^)	Low suitable area (×10^4^ km^2^)	Medium suitable area (×10^4^ km^2^)	High suitable area (×10^4^ km^2^)
Current		40.94	19.80	12.01	9.13
SSP1-2.6	2021-2040	37.20	18.56	11.20	7.44
	2061-2080	35.71	17.91	10.48	7.32
SSP2-4.5	2021-2040	39.34	20.20	11.38	7.76
	2061-2080	34.22	18.11	9.72	6.39
SSP5-8.5	2021-2040	37.60	18.91	10.98	7.71
	2061-2080	27.55	14.66	8.21	4.68

#### 3.3.2 Potential distribution of *K. evelyniana* under future climate scenarios

**Figure 6:**
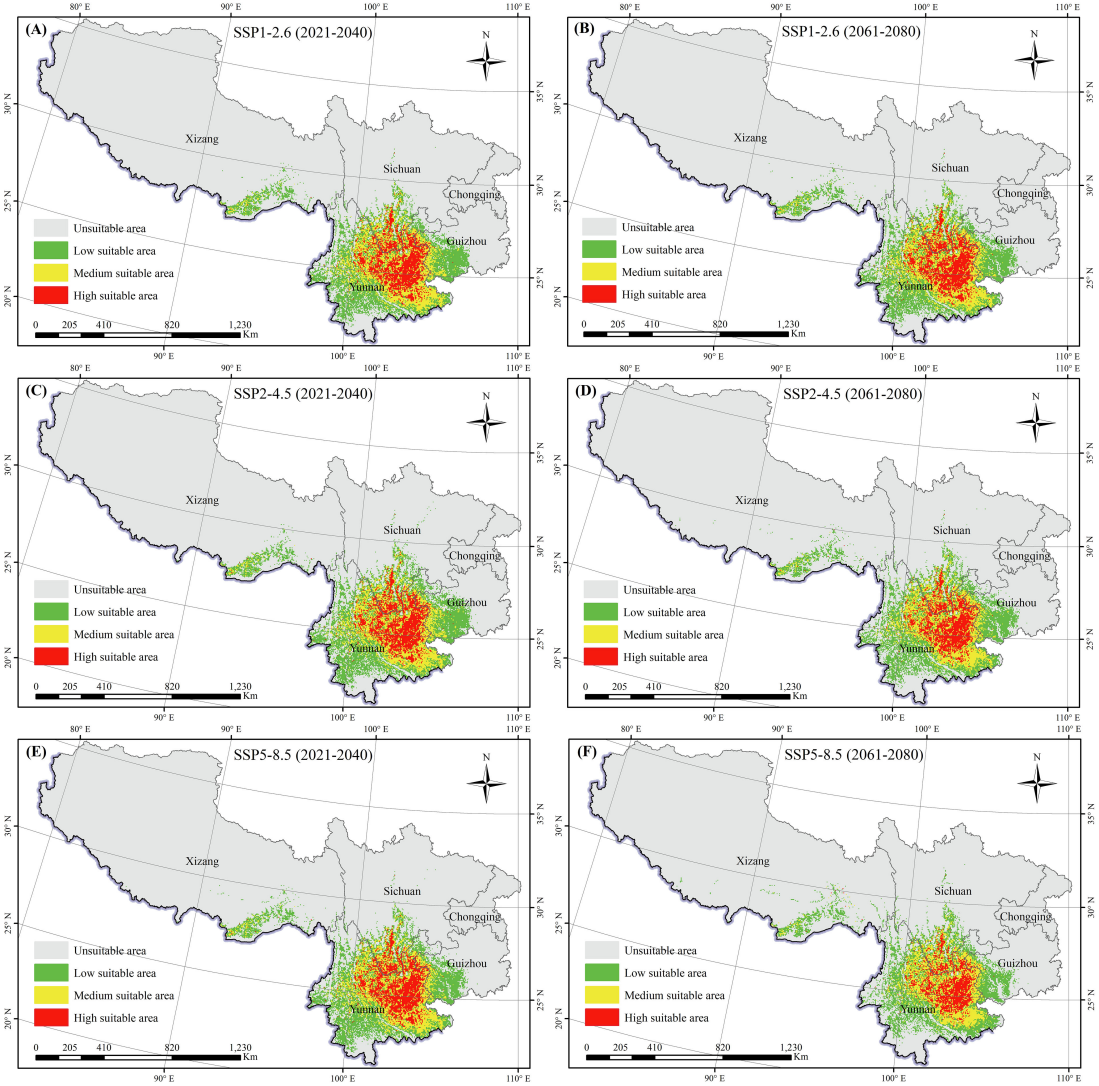
Potential distribution of *K. evelyniana* under future climate scenarios: **(A, C, E)** represent scenarios SSP1-2.6, SSP2-4.5, and SSP5-8.5 for the period 2021-2040; **(B, D, F)** represent scenarios SSP1-2.6, SSP2-4.5, and SSP5-8.5 for the period 2061-2080.

The Maxent model was implemented to predict the suitable areas for *K. evelyniana* under various future climate scenarios, and the results are shown in **Table 2** and **Figure 6**. Compared with the current climate conditions, the future scenarios indicated a decreasing trend in suitable areas for *K. evelyniana*. Under SSP5-8.5 scenarios, the dominant significant reduction was observed during the 2061–2080 period, with a 33% decline in the suitable area.

**Figure 7:**
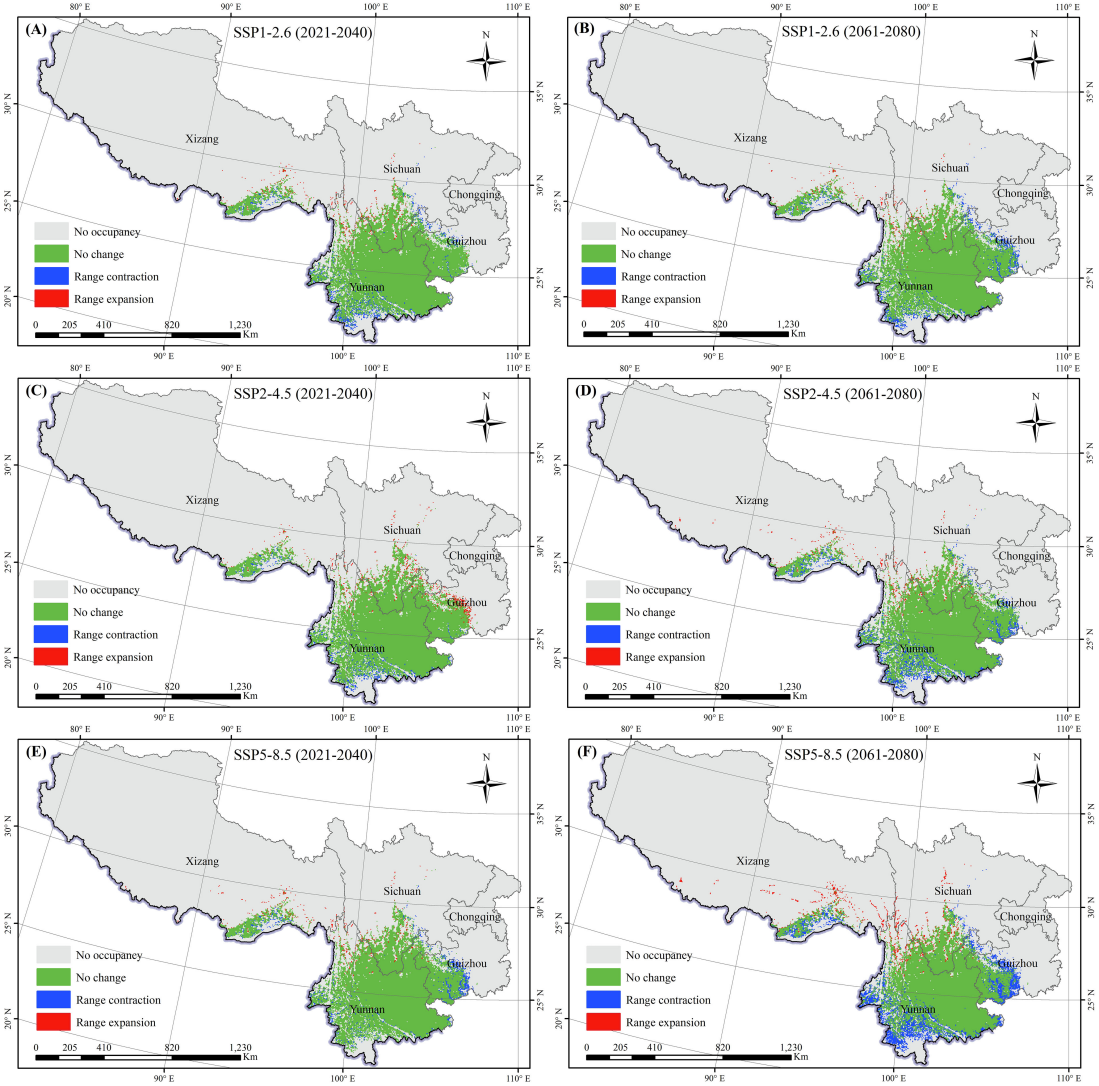
Changes in the potential distribution of *K. evelyniana* under future climate scenarios: **(A, C, E)** represent scenarios SSP1-2.6, SSP2-4.5, and SSP5-8.5 for the period 2021-2040; **(B, D, F)** represent scenarios SSP1-2.6, SSP2-4.5, and SSP5-8.5 for the period 2061-2080.

To describe the spatial variations in suitable areas for *K. evelyniana* due to climate change, the binary maps of the current climate scenarios were compared with those from the 2030s and 2070s under various future climate scenarios (**Table 3**; **Figure 7**). The results indicated that suitable areas for *K. evelyniana* expanded and contracted across different future periods, whereas the contraction areas consistently exceeded the expansion areas, reducing the total suitable area. During 2021–2040, the changes in the area under SSP5-8.5, SSP2-4.5, and SSP1-2.6 were -1.19×10^4^, -0.09×10^4^, and -1.44×10^4^ km², respectively, with the smallest decrease occurring under SSP2-4.5. By 2061-2080, the decline in suitable area intensified, with the reductions of 5.74×10^4^, 2.82×10^4^, and 2.24×10^4^ km^2^ under SSP5-8.5, SSP2-4.5, and SSP1-2.6, respectively.

**Table 3:** Changes in suitable area for *K. evelyniana* under different future climate scenarios compared to current conditions.

Scenarios	Period	Total suitable area (×10^4^ km^2^)	Range expansion (×10^4^ km^2^)	No change (×10^4^ km^2^)	Range contraction (×10^4^ km ^2^)
SSP1-2.6	2021-2040	37.20	0.87	38.64	2.31
	2061-2080	35.71	0.76	37.95	3.00
SSP2-4.5	2021-2040	39.34	1.42	39.43	1.51
	2061-2080	34.22	1.09	37.04	3.91
SSP5-8.5	2021-2040	37.60	0.96	38.79	2.15
	2061-2080	27.55	1.92	33.29	7.66

#### 3.3.3 Centroid migration of *K. evelyniana* under future climate scenarios

The shape of the suitable area for *K. evelyniana* was irregular. To measure the changes in its distribution, the centroid was defined, and its migration was calculated and analyzed under different scenarios (**Figure 5B**). Under the current conditions, the centroid was located in Yuanmou County, Yunnan Province (101.927E, 25.517N). In the future climate scenarios, the centroid gradually migrated to the northeast, north, and northwest, with migration distances ranging from 8.33 to 33.40 km. Additionally, for the same climate scenario, the migration distance between 2061 and 2080 is greater than that between 2021 and 2040.

## 4 Discussion

### 4.1 Accuracy and reliability of the *K. evelyniana* Maxent model

This study utilized the Maxent model to predict the potential distribution of *K. evelyniana* in southwest China. After performing 10-fold cross-validation on the environmental factors and distribution data, the model achieved an AUC value of 0.946 (± 0.011), demonstrating its excellent predictive ability. This was consistent with AUC values between 0.9 and 0.98 reported in related studies on conifer species, affirming the accuracy and reliability of the results (Zhao et al., 2021b; Duan et al., 2022; Feng et al., 2023; He et al., 2023). According to the model predictions, *K. evelyniana* was primarily distributed in Yunnan Province, with additional suitable areas in western and southwestern Guizhou, southwestern Sichuan, and the southeastern Xizang Autonomous Region (**Figure 5A**). The predicted distribution of *K. evelyniana* was closely aligned with its description in the Flora of China (Editorial Committee of flora of China, Chinese Academy of Sciences, 2004). To prevent overfitting and improve the model’s migration capability, this study, following similar research (Li et al., 2020; Shi et al., 2024; Zhao et al., 2021b), adjusted the Maxent model parameters and analyzed the model complexity using the ENMeval package. The parameter with the lowest complexity was selected to run the Maxent model to ensure more accurate predictions. With the model parameters set to FC=LQ and RM=0.5, the Maxent model produced the smallest delta.AICc value of 0 (**Figure 2A**), indicating the lowest complexity. In summary, the reliability and accuracy of the *K. evelyniana* Maxent model were validated using effective methods, parameter optimization, and robust results.

### 4.2 Dominant environmental factors influencing the *K. evelyniana* distribution

Based on a comprehensive analysis of percent contribution, permutation importance, regularization training gain only with this variable and regularization training gain without this variable (**Table 1**), temperature was identified as the most crucial environmental variable affecting the potential distribution of *K. evelyniana*, with a cumulative contribution of 77.8%. Similar results have been demonstrated for other conifer species. For instance, Zhao et al. (2021b) suggested that temperature was the primary factor affecting the distribution of *Cunninghamia lanceolata* (Lamb.), contributing 64.24%, whereas Feng et al. (2023) identified Bio4 as a key factor for *Pinus yunnanensis* Franch. Zhang et al. (2023) also reported that two of the three most significant factors influencing the distribution of *Keteleeria davidiana* (C. E. Bertrand) Beissn were temperature-related. *K. evelyniana* exhibited the most sensitivity to min temperature of coldest month (Bio6), indicating that extremely low temperatures may be a critical limiting factor for its distribution. Low temperatures can cause frost damage, impair membrane function, and reduce photosynthesis, thereby significantly hindering plant growth (Ladwig et al., 2016). In this study, the temperature seasonality (Bio4) range of 370–550 was found to favor the expansion of *K. evelyniana*’s suitable areas, as temperature seasonality plays a vital role in plant growth, development, and flowering, with many species requiring a warm-cold-warm cycle to complete their annual life cycle (Khodorova and Boitel-Conti, 2013).

Soil plays a crucial role in regulating nutrients necessary for plant growth, making it essential for plant development (Dighton, 2014). In this study, Sand, Soc, and Bdod were found to significantly influence the potential distribution of *K. evelyniana*, with a cumulative contribution of 20.5%. Although elevation had a smaller contribution to the final model, its importance was highlighted in the regularization training gain without this variable (**Table 1**). Elevation can primarily affect species distribution patterns through its indirect effect on temperature and precipitation (Yin et al., 2023).

### 4.3 Potential distribution change and centroid migration of *K. evelyniana* under future climate scenarios

The low, medium, and high suitability areas for *K. evelyniana* exhibited the decreasing trends (**Table 2**; **Figure 6**). The most significant decline occurred under the SSP5-8.5 scenario during 2061–2080, with a 33% reduction in the suitable area. In the same scenario, the decrease in suitable areas was greater in 2061–2080 than in 2021–2040. Additionally, for both periods, the reduction was smaller in the low-emission scenario and larger in the high-emission scenario. Under the future conditions, the distribution of *K. evelyniana* could demonstrate not only a reduction in suitable areas but also significant shifts in spatial patterns, particularly at the edges of suitable regions (**Figure 7**). In high emission scenarios, marked by dramatic and irregular changes in temperature and precipitation, along with more frequent extreme events, the impact on *K. evelyniana* could be substantial, leading to increased habitat fragmentation (Fan et al., 2021; Zhang et al., 2021).

Under climate change scenarios, shifts in the distribution centroid of *K. evelyniana*’s suitable area can reflect its movement in both distance and direction. Compared to current climate conditions, the centroid was projected to move to higher latitudes in the north, northeast, and northwest under various greenhouse gas emission scenarios during 2021–2040 and 2061–2080, with the longest migration distance reaching 33.40 km (**Figure 5B**). This is consistent with previous studies indicating that a warming climate prompts plant migration to higher latitudes and altitudes (Chi et al., 2023; Shi et al., 2023; Zhou et al., 2023a). Such shifts in species distribution may be an adaptive response to global warming, because rising temperatures render the original habitats too warm, forcing them to move to cooler areas. This migration helps species maintain the temperature range and ecological niche essential for their survival (Chen et al., 2011; Dullinger et al., 2012).

### 4.4 Future conservation strategies and research perspectives for the *K. evelyniana*

Several strategies should be implemented to alleviate the impact of climate change on the *K. evelyniana* populations. For areas lost due to climate change, it is necessary to re-evaluate conservation strategies and adopt measures such as *ex-situ* conservation and germplasm preservation (Wang et al., 2024). Maintaining suitable areas is also crucial, as these regions serve as refuges and shelters for future climate change. Prioritizing the conservation and management of *K. evelyniana* habitats is essential. This involves, first, protecting existing habitats to provide a stable base for species survival, second, restoring and enhancing degraded habitats to improve their resilience to climate change, and third, developing adaptive management strategies (Guignabert et al., 2024), such as adjusting conservation objectives and methods to address new challenges posed by climate change. For newly suitable areas, it is crucial to establish ecological corridors to facilitate species migration and gene flow and to consider planned assisted translocations (Ding, 2023; Twardek et al., 2023).

Studies have shown that species misidentification (Phillips et al., 2009), sampling bias (Leitão et al., 2011; Beck et al., 2014), temporal or spatial bias (Graham et al., 2008; Meyer et al., 2015) and sampling strategy (Hirzel and Guisan, 2002) in species occurrence data can lead to uncertainty in species distribution models. In addition, the quality of environmental data (Graham et al., 2008), model assumptions (Chen et al., 2019) and biological interactions (Wisz et al., 2013) also contribute to uncertainty in these models. Future research should focus on improving the quality of species distribution data, including enhancing species identification methods, optimizing sampling design, and filling data gaps. At the same time, the accuracy of environmental data should be improved, and their uncertainty taken into account. In terms of model development, it is recommended to adopt multi-model comparisons and dynamic simulation approaches, incorporating biological interactions to improve ecological rationality.

Örücü et al. (2023) found that land-use change affects species’ migration speed and distribution range by altering habitat structure and availability, with more pronounced impacts in tropical regions. Population density was positively correlated with the distribution probability of *Pinus massoniana*, indicating that *Pinus massoniana* was more widely distributed in densely populated areas (He et al., 2023). The construction of large-scale ecological engineering projects may indirectly affect species distribution ranges by changing local climate conditions (Wang and Guan, 2023). Only topographic, soil, and bioclimatic factors were considered, excluding important factors such as human disturbance, land use and cover changes. Future research should incorporate more environmental variables and continue to optimize the model to enhance its predictive accuracy.

## 5 Conclusion

Based on 33 environmental variables and 221 distribution points, this study utilized the Maxent model to simulate suitable areas for *K. evelyniana* in southwest China under current and future climate scenarios, while analyzing the dominant environmental factors that could affect its distribution. The total suitable area for *K. evelyniana* was 40.94×10^4^ km^2^, with 68% concentrated in Yunnan Province. Other suitable areas were distributed in western and southwestern Guizhou, southwestern Sichuan, and southeastern Xizang Autonomous Region, with no suitable areas in the Chongqing Municipality. Under the future scenarios, the suitable area is projected to gradually decrease, with a 33% reduction during 2061–2080 under the SSP5-8.5. Currently, the centroid was located in Yuanmou County, Yunnan Province (101.927E, 25.517N), but it is expected to shift northeast, north, and northwest, with migration distances ranging from 8.33 to 33.40 km under different climate scenarios. Temperature (Bio4 and Bio6), soil, and elevation jointly influenced species distribution, with temperature having the most significant effect (77.8%). Our findings provide a theoretical foundation for conservation, ecological restoration, and sustainable use of *K. evelyniana*.

## Data Availability

Publicly available datasets were analyzed in this study. This data can be found here: Global Biodiversity Information Facility (GBIF, https://www.gbif.org/), the Chinese Virtual Herbarium (CVH, https://www.cvh.ac.cn/), and the literature (Tang et al., 2017; Du, 2022).

## References

[B1] Beck J. Böller M. Erhardt A. Schwanghart W. (2014). Spatial bias in the GBIF database and its effect on modeling species’ geographic distributions. Ecol. Inf. 19, 10–15. doi: 10.1016/j.ecoinf.2013.11.002

[B2] Bellard C. Bertelsmeier C. Leadley P. Thuiller W. Courchamp F. (2012). Impacts of climate change on the future of biodiversity. Ecol. Lett. 15, 365–377. doi: 10.1111/j.1461-0248.2011.01736.x 22257223 PMC3880584

[B3] Boussouf S. Fernández T. Hart A. B. (2023). Landslide susceptibility mapping using maximum entropy (MaxEnt) and geographically weighted logistic regression (GWLR) models in the Río Aguas catchment (Almería, SE Spain). Nat. Hazards. 117, 207–235. doi: 10.1007/s11069-023-05857-7

[B4] Brown J. L. Bennett J. R. French C. M. (2017). SDMtoolbox 2.0: the next generation Python-based GIS toolkit for landscape genetic, biogeographic and species distribution model analyses. PeerJ 5, e4095. doi: 10.7717/peerj.4095 29230356 PMC5721907

[B5] Calvin K. Dasgupta D. Krinner G. Mukherji A. Thorne P. W. Trisos C.. (2023). IPCC 2023: Climate Change 2023: Synthesis Report. Contribution of Working Groups I, II and III to the Sixth Assessment Report of the Intergovernmental Panel on Climate Change (IPCC, Geneva, Switzerland: Intergovernmental Panel on Climate Change (IPCC). Available online at: https://www.ipcc.ch/report/ar6/syr/ (Accessed June 26, 2024).

[B6] Chen X. Dimitrov N. B. Meyers L. A. (2019). Uncertainty analysis of species distribution models. PloS One 14, e0214190. doi: 10.1371/journal.pone.0214190 31120909 PMC6533036

[B7] Chen I.-C. Hill J. K. Ohlemüller R. Roy D. B. Thomas C. D. (2011). Rapid range shifts of species associated with high levels of climate warming. Science 333, 1024–1026. doi: 10.1126/science.1206432 21852500

[B8] Chi Y. Wang G. G. Zhu M. Jin P. Hu Y. Shu P.. (2023). Potentially suitable habitat prediction of *Pinus massoniana* Lamb. in China under climate change using Maxent model. Front. For. Glob. Change 6. doi: 10.3389/ffgc.2023.1144401

[B9] Deng Z. Xia X. Zhang M. Chen X. Ding X. Zhang B.. (2024). Predicting the spatial distribution of the mangshan pit viper (*Protobothrops mangshanensis*) under climate change scenarios using MaxEnt modeling. Forests 15, 723. doi: 10.3390/f15040723

[B10] Dieleman C. M. Branfireun B. A. McLaughlin J. W. Lindo Z. (2015). Climate change drives a shift in peatland ecosystem plant community: Implications for ecosystem function and stability. Global Change Biol. 21, 388–395. doi: 10.1111/gcb.12643 24957384

[B11] Dighton J. (2014). “Introduction: Soils and their promotion of plant growth,” in Interactions in Soil: Promoting Plant Growth. Eds. Dighton J. Krumins J. A. (Springer Netherlands, Dordrecht), 1–26. doi: 10.1007/978-94-017-8890-8_1

[B12] Ding G. (2023). Protecting and constructing ecological corridors for biodiversity conservation: A framework that integrates landscape similarity assessment. Appl. Geogr. 160, 103098. doi: 10.1016/j.apgeog.2023.103098

[B13] Du M. (2022). Forest types, structure and regenerationdynamics of *Keteleeria evelyniana* in China (Kunming Yunnan: Yunnan University).

[B14] Duan X. Li J. Wu S. (2022). MaxEnt modeling to estimate the impact of climate factors on distribution of *Pinus densiflora* . Forests 13, 402. doi: 10.3390/f13030402

[B15] Dullinger S. Gattringer A. Thuiller W. Moser D. Zimmermann N. E. Guisan A.. (2012). Extinction debt of high-mountain plants under twenty-first-century climate change. Nat. Clim Change 2, 619–622. doi: 10.1038/nclimate1514

[B16] Editorial Committee of flora of China, Chinese Academy of Sciences (2004). Flora of China (Beijing: Science Press).

[B17] Elith J. Graham C. H. Anderson R. P. Dudík M. Ferrier S. Guisan A.. (2006). Novel methods improve prediction of species’ distributions from occurrence data. Ecography 29, 129–151. doi: 10.1111/j.2006.0906-7590.04596.x

[B18] Elith J. Leathwick J. R. (2009). Species distribution models: Ecological explanation and prediction across space and time. Annu. Rev. Ecol. Evol. Syst. 40, 677–697. doi: 10.1146/annurev.ecolsys.110308.120159

[B19] Elith J. Phillips S. J. Hastie T. Dudík M. Chee Y. E. Yates C. J. (2011). A statistical explanation of MaxEnt for ecologists: Statistical explanation of MaxEnt. Diversity Distrib. 17, 43–57. doi: 10.1111/j.1472-4642.2010.00725.x

[B20] Fan X. Miao C. Duan Q. Shen C. Wu Y. (2021). Future climate change hotspots under different 21st century warming scenarios. Earth’s. Future 9, e2021EF002027. doi: 10.1029/2021EF002027

[B21] Feeley K. J. Bravo-Avila C. Fadrique B. Perez T. M. Zuleta D. (2020). Climate-driven changes in the composition of New World plant communities. Nat. Clim. Change 10, 965–970. doi: 10.1038/s41558-020-0873-2

[B22] Feng J. Wang B. Xian M. Zhou S. Huang C. Cui X. (2023). Prediction of future potential distributions of *Pinus yunnanensis* varieties under climate change. Front. For. Glob. Change 6. doi: 10.3389/ffgc.2023.1308416

[B23] Fick S. E. Hijmans R. J. (2017). WorldClim 2: new 1-km spatial resolution climate surfaces for global land areas. Int. J. Climatol. 37, 4302–4315. doi: 10.1002/joc.5086

[B24] Fu Z. Zhang Y. Tan N. Chu H. Ji C. (2008). Chemical constituents of *Keteleeria evelyniana* . Natural Product. Res. Dev., 257–261 + 277. doi: 10.16333/j.1001-6880.2008.02.022

[B25] Gao X. Liu J. Huang Z. (2022). The impact of climate change on the distribution of rare and endangered tree *Firmiana kwangsiensis* using the Maxent modeling. Ecol. Evol. 12, e9165. doi: 10.1002/ece3.9165 35919389 PMC9336174

[B26] Ge Z.-W. Smith M. E. Zhang Q.-Y. Yang Z. L. (2012). Two species of the Asian endemic genus *Keteleeria* form ectomycorrhizas with diverse fungal symbionts in southwestern China. Mycorrhiza 22, 403–408. doi: 10.1007/s00572-011-0411-1 21997220

[B27] Graham C. H. Elith J. Hijmans R. J. Guisan A. Townsend Peterson A. Loiselle B. A.. (2008). The influence of spatial errors in species occurrence data used in distribution models. J. Appl. Ecol. 45, 239–247. doi: 10.1111/j.1365-2664.2007.01408.x

[B28] Guignabert A. Jonard M. Messier C. Andr´e F. de Coligny F. Doyon F.. (2024). Adaptive forest management improves stand-level resilience of temperate forests under multiple stressors. Sci. Total. Environ. 948, 174168. doi: 10.1016/j.scitotenv.2024.174168 38942315

[B29] He W. J. Fu Z. H. Han H. J. Yan H. Zeng G. Z. Ji C. J.. (2011). Benzoic acid allopyranosides and lignan glycosides from the twigs of *Keteleeria evelyniana* . Z. Naturforschung. B. 66, 733–739. doi: 10.1515/znb-2011-0715

[B30] He Y. Ma J. Chen G. (2023). Potential geographical distribution and its multi-factor analysis of *Pinus massoniana* in China based on the maxent model. Ecol. Indic. 154, 110790. doi: 10.1016/j.ecolind.2023.110790

[B31] Hirzel A. Guisan A. (2002). Which is the optimal sampling strategy for habitat suitability modelling. Ecol. Model. 157, 331–341. doi: 10.1016/S0304-3800(02)00203-X

[B32] IPCC (2023). “Climate Change 2023: Synthesis Report. Contribution of Working Groups I, II and III to the Sixth Assessment Report of the Intergovernmental Panel on Climate Change,” eds. Core Writing Team, H. Lee, and J. Romero (Geneva, Switzerland: IPCC).

[B33] Kass J. M. Muscarella R. Galante P. J. Bohl C. L. Pinilla-Buitrago G. E. Boria R. A.. (2021). ENMeval 2.0: Redesigned for customizable and reproducible modeling of species’ niches and distributions. Methods Ecol. Evol. 12, 1602–1608. doi: 10.1111/2041-210X.13628

[B34] Khodorova N. Boitel-Conti M. (2013). The role of temperature in the growth and flowering of geophytes. Plants 2, 699–711. doi: 10.3390/plants2040699 27137399 PMC4844387

[B35] Kuprin A. Shevchenko N. Baklanova V. (2024). Modelling distribution of an endangered longhorn beetle, *Callipogon relictus* (Coleoptera: Cerambycidae), in northeast asia. Forests 15, 598. doi: 10.3390/f15040598

[B36] Ladwig L. M. Ratajczak Z. R. Ocheltree T. W. Hafich K. A. Churchill A. C. Frey S. J. K.. (2016). Beyond arctic and alpine: the influence of winter climate on temperate ecosystems. Ecology 97, 372–382. doi: 10.1890/15-0153.1 27145612

[B37] Lawlor J. A. Comte L. Grenouillet G. Lenoir J. Baecher J. A. Bandara R. M. W. J.. (2024). Mechanisms, detection and impacts of species redistributions under climate change. Nat. Rev. Earth Environ. 5, 351–368. doi: 10.1038/s43017-024-00527-z

[B38] Leitão P. J. Moreira F. Osborne P. E. (2011). Effects of geographical data sampling bias on habitat models of species distributions: A case study with steppe birds in southern Portugal. Int. J. Geographical. Inf. Sci. 25, 439–454. doi: 10.1080/13658816.2010.531020

[B39] Li J. Fan G. He Y. (2020). Predicting the current and future distribution of three Coptis herbs in China under climate change conditions, using the MaxEnt model and chemical analysis. Sci. Total. Environ. 698, 134141. doi: 10.1016/j.scitotenv.2019.134141 31505366

[B40] Li X. Zhao A. Dang C. Peng M. (2013). A study on the structure and regeneration of *Keteleeria evelyniana* mixed forest in Xishan,Kunming. J. Yunnan. Univ.: Natural Sci. Edition. 35, 549–557.

[B41] Low B. W. Zeng Y. Tan H. H. Yeo D. C. J. (2021). Predictor complexity and feature selection affect Maxent model transferability: Evidence from global freshwater invasive species. Diversity Distrib. 27, 497–511. doi: 10.1111/ddi.13211

[B42] Meyer C. Weigelt P. Kreft H. (2015). Multidimensional biases, gaps and uncertainties in global plant occurrence information. 19(8), 992–1006. doi: 10.7287/peerj.preprints.1326v2 27250865

[B43] Miller J. (2010). Species distribution modeling. Geogr. Compass. 4, 490–509. doi: 10.1111/j.1749-8198.2010.00351.x

[B44] Örücü Ö. K. Azadi H. Arslan E. S. Kamer Aksoy Ö. Choobchian S. Nooghabi S. N.. (2023). Predicting the distribution of European Hop Hornbeam: application of MaxEnt algorithm and climatic suitability models. Eur. J. For. Res. 142, 579–591. doi: 10.1007/s10342-023-01543-2

[B45] Paz-Kagan T. Chang J. G. Shoshany M. Sternberg M. Karnieli A. (2021). Assessment of plant species distribution and diversity along a climatic gradient from Mediterranean woodlands to semi-arid shrublands. GISci. Remote Sens. 58, 929–953. doi: 10.1080/15481603.2021.1953770

[B46] Pearson R. G. Raxworthy C. J. Nakamura M. Peterson A. T. (2007). Predicting species distributions from small numbers of occurrence records: a test case using cryptic geckos in Madagascar. J. Biogeogr. 34, 102–117. doi: 10.1111/j.1365-2699.2006.01594.x

[B47] Phillips S. J. Dudík M. Elith J. Graham C. H. Lehmann A. Leathwick J.. (2009). Sample selection bias and presence-only distribution models: Implications for background and pseudo-absence data. Ecol. Appl. 19, 181–197. doi: 10.1890/07-2153.1 19323182

[B48] Phillips S. J. Dudık M. (2008). Modeling of species distributions with Maxent: new extensions and a comprehensive evaluation. Ecography 31, 161–175. doi: 10.1111/j.0906-7590.2008.5203.x

[B49] Poggio L. De Sousa L. M. Batjes N. H. Heuvelink G. B. M. Kempen B. Ribeiro E.. (2021). SoilGrids 2.0: producing soil information for the globe with quantified spatial uncertainty. SOIL 7, 217–240. doi: 10.5194/soil-7-217-2021

[B50] Qasimi A. B. Isazade V. Berndtsson R. (2024). Flood susceptibility prediction using MaxEnt and frequency ratio modeling for Kokcha River in Afghanistan. Nat. Hazards. 120, 1367–1394. doi: 10.1007/s11069-023-06232-2

[B51] Ruiz-Labourdette D. Nogués-Bravo D. Ollero H. S. Schmitz M. F. Pineda F. D. (2012). Forest composition in Mediterranean mountains is projected to shift along the entire elevational gradient under climate change. J. Biogeogr. 39, 162–176. doi: 10.1111/j.1365-2699.2011.02592.x

[B52] Shi X. Wang J. Zhang L. Chen S. Zhao A. Ning X.. (2023). Prediction of the potentially suitable areas of *Litsea cubeba* in China based on future climate change using the optimized MaxEnt model. Ecol. Indic. 148, 110093. doi: 10.1016/j.ecolind.2023.110093 PMC10585429 37869439

[B53] Shi J. Xia M. He G. Gonzalez N. C. T. Zhou S. Lan K.. (2024). Predicting *Quercus gilva* distribution dynamics and its response to climate change induced by GHGs emission through MaxEnt modeling. J. Environ. Manage. 357, 120841. doi: 10.1016/j.jenvman.2024.120841 38581898

[B54] Soilhi Z. Sayari N. Benalouache N. Mekki M. (2022). Predicting current and future distributions of *Mentha pulegium* L. @ in Tunisia under climate change conditions, using the MaxEnt model. Ecol. Inf. 68, 101533. doi: 10.1016/j.ecoinf.2021.101533

[B55] Sorbe F. Gränzig T. Förster M. (2023). Evaluating sampling bias correction methods for invasive species distribution modeling in Maxent. Ecol. Inf. 76, 102124. doi: 10.1016/j.ecoinf.2023.102124

[B56] Swets J. A. (1988). Measuring the accuracy of diagnostic systems. Science 240, 1285–1293. doi: 10.1126/science.3287615 3287615

[B57] Tang C. Q. Du M. Wang H. Shi Y. Zeng J. Xiao S.. (2024). An unprotected vulnerable relict subtropical conifer-*Keteleeria evelyniana*: Its forests, populations, growth and endangerment by invasive alien plant species in China. Plant Diversity 24, 648–660. doi: 10.1016/j.pld.2024.02.006 PMC11403115 39290888

[B58] Tang S. Yang J. Yuan M. Li S. Zhou Q. (2017). *Keteleeria evelyniana*: a newly recorded species of Pinaceae in Guiyang. Guizhou. Sci. 35, 19–20.

[B59] Thomas C. D. Cameron A. Green R. E. Bakkenes M. Beaumont L. J. Collingham Y. C.. (2004). Extinction risk from climate change. Nature 427, 145–148. doi: 10.1038/nature02121 14712274

[B60] Thuiller W. Georges D. Gueguen M. Engler R. Breiner F. Lafourcade B.. (2012). biomod2: Ensemble platform for species distribution modeling 4.2-6–4.2-2. doi: 10.32614/CRAN.package.biomod2

[B61] Twardek W. M. Taylor J. J. Rytwinski T. Aitken S. N. MacDonald A. Bogaert R. V.. (2023). The application of assisted migration as a climate change adaptation tactic: An evidence map and synthesis. Biol. Conserv. 280, 109932. doi: 10.1016/j.biocon.2023.109932

[B62] Wang M. Guan Q. (2023). Prediction of potential suitable areas for *Broussonetia papyrifera* in China using the MaxEnt model and CIMP6 data. J. Plant Ecol. 16, rtad006. doi: 10.1093/jpe/rtad006

[B63] Wang S. Li L. Liu C. Jiang R. Huang X. He J.. (2022). Effects of exogenous hormone and substrate on seed germination of *Keteleeria evelyniana* . Chin. Agric. Sci. Bull. 38, 15–21. doi: 10.11924/j.issn.1000-6850.casb2021-0622

[B64] Wang Z. Wang T. Zhang X. Wang J. Yang Y. Sun Y.. (2024). Biodiversity conservation in the context of climate change: Facing challenges and management strategies. Sci. Total. Environ. 937, 173377. doi: 10.1016/j.scitotenv.2024.173377 38796025

[B65] Warren D. L. Seifert S. N. (2011). Ecological niche modeling in Maxent: the importance of model complexity and the performance of model selection criteria. Ecol. Appl. 21, 335–342. doi: 10.1890/10-1171.1 21563566

[B66] Warren D. L. Wright A. N. Seifert S. N. Shaffer H. B. (2014). Incorporating model complexity and spatial sampling bias into ecological niche models of climate change risks faced by 90 C alifornia vertebrate species of concern. Diversity Distrib. 20, 334–343. doi: 10.1111/ddi.12160

[B67] Wei X. Xu D. Liu Q. Wu Y. Zhuo Z. (2024). Predicting the potential distribution range of *Batocera horsfieldi* under CMIP6 climate change using the MaxEnt model. J. Econ. Entomol. 117, 187–198. doi: 10.1093/jee/toad209 38007398

[B68] Wilson R. J. Gutiérrez D. Gutiérrez J. Monserrat V. J. (2007). An elevational shift in butterfly species richness and composition accompanying recent climate change. Global Change Biol. 13, 1873–1887. doi: 10.1111/j.1365-2486.2007.01418.x

[B69] Wisz M. S. Pottier J. Kissling W. D. Pellissier L. Lenoir J. Damgaard C. F.. (2013). The role of biotic interactions in shaping distributions and realised assemblages of species: Implications for species distribution modelling. Biol. Rev. 88, 15–30. doi: 10.1111/j.1469-185X.2012.00235.x 22686347 PMC3561684

[B70] Yan X. Wang S. Duan Y. Han J. Huang D. Zhou J. (2021). Current and future distribution of the deciduous shrub *Hydrangea macrophylla* in China estimated by MaxEnt. Ecol. Evol. 11, 16099–16112. doi: 10.1002/ece3.8288 34824814 PMC8601876

[B71] Yin D. Gou X. Yang H. Wang K. Liu J. Zhang Y.. (2023). Elevation-dependent tree growth response to recent warming and drought on eastern Tibetan Plateau. Clim. Change 176, 77. doi: 10.1007/s10584-023-03542-z

[B72] Zhang Q. Shen X. Jiang X. Fan T. Liang X. Yan W. (2023). MaxEnt modeling for predicting suitable habitat for endangered tree *Keteleeria davidiana* (Pinaceae) in China. Forests 14, 394. doi: 10.3390/f14020394

[B73] Zhang G. Zeng G. Yang X. Jiang Z. (2021). Future changes in extreme high temperature over China at 1.5°C–5°C global warming based on CMIP6 simulations. Adv. Atmos. Sci. 38, 253–267. doi: 10.1007/s00376-020-0182-8

[B74] Zhao G. Cui X. Sun J. Li T. Wang Q. Ye X.. (2021a). Analysis of the distribution pattern of Chinese Ziziphus jujuba under climate change based on optimized biomod2 and MaxEnt models. Ecol. Indic. 132, 108256. doi: 10.1016/j.ecolind.2021.108256

[B75] Zhao Y. Deng X. Xiang W. Chen L. Ouyang S. (2021b). Predicting potential suitable habitats of Chinese fir under current and future climatic scenarios based on Maxent model. Ecol. Inf. 64, 101393. doi: 10.1016/j.ecoinf.2021.101393

[B76] Zhao Z. Xiao N. Shen M. Li J. (2022). Comparison between optimized MaxEnt and random forest modeling in predicting potential distribution: A case study with Quasipaa boulengeri in China. Sci. Total. Environ. 842, 156867. doi: 10.1016/j.scitotenv.2022.156867 35752245

[B77] Zhou W. Li B. Xu H. Liang Z. Lu X. Yang L.. (2023b). Potential distribution of two economic laver species-*Neoporphyra haitanensis* and *Neopyropia yezoensis* under climate change based on MaxEnt prediction and phylogeographic profiling. Ecol. Indic. 150, 110219. doi: 10.1016/j.ecolind.2023.110219

[B78] Zhou D. Li L. Zhu C. Gu M. Zhang H. Li X.. (2023a). Effect of melatonin on seed germination and seedling growth and physiological properties of *Keteleeria evelyniana* . J. Sichuan. Agric. Univ. 41, 249–256. doi: 10.16036/j.issn.1000-2650.202209188

