# Supplementary material for: Assessing the impact of climate change on the potential distribution of Keteleeria evelyniana Mast. in southwest China: a Maxent modeling approach
Source: Front Plant Sci. 2025 Apr 25;16:1561031. doi: 10.3389/fpls.2025.1561031 (PMC12090538; doi:10.3389/fpls.2025.1561031)
Supplement: Supplementary file 1 [file DataSheet1.docx]

**Appendix**

**Table S1** Environment variables

| **Variable type** | **Environment variables** | **Variable description** | **Unit** | **Contribution rate of initial operation /%** |
| --- | --- | --- | --- | --- |
| Bioclimatic | Bio1 | Annual mean temperature | ℃ | 0.5 |
|  | Bio2 | Mean diurnal range (Mean of monthly (max temp - min temp)) | ℃ | 0.4 |
|  | Bio3 | Isothermality (BIO2/BIO7) (×100) | - | 3.9 |
|  | Bio4 | Temperature seasonality (standard deviation ×100) | - | 38.9 |
|  | Bio5 | Max temperature of warmest month | ℃ | 0.2 |
|  | Bio6 | Min temperature of coldest month | ℃ | 24.6 |
|  | Bio7 | Temperature annual range (BIO5-BIO6) | ℃ | 0.5 |
|  | Bio8 | Mean temperature of wettest quarter | ℃ | 0.2 |
|  | Bio9 | Mean temperature of driest quarter | ℃ | 0.9 |
|  | Bio10 | Mean temperature of warmest quarter | ℃ | 0.1 |
|  | Bio11 | Mean temperature of coldest quarter | ℃ | 0.6 |
|  | Bio12 | Annual precipitation | mm | 0.4 |
|  | Bio13 | Precipitation of wettest month | mm | 0.2 |
|  | Bio14 | Precipitation of driest month | mm | 0.1 |
|  | Bio15 | Precipitation seasonality (Coefficient of variation) | % | 0.2 |
|  | Bio16 | Precipitation of wettest quarter | mm | 0.1 |
|  | Bio17 | Precipitation of driest quarter | mm | 0.3 |
|  | Bio18 | Precipitation of warmest quarter | mm | 0.1 |
|  | Bio19 | Precipitation of coldest quarter | mm | 0.4 |
| Topographic | Elev | Elevation | m | 11.8 |
|  | Aspect | Aspect | ° | 0.8 |
|  | Slope | Slope | ° | 0.4 |
| Soil | Bdod | Bulk density of the fine earth fraction | kg/dm³ | 1.6 |
|  | Cec | Cation exchange capacity of the soil | cmol(c)/kg | 0.3 |
|  | Cfvo | Volumetric fraction of coarse fragments (> 2 mm) | cm^3^/100cm^3^ (vol%) | 0.4 |
|  | Clay | Proportion of clay particles (< 0.002 mm) in the fine earth fraction | g/100g (%) | 0.6 |
|  | Nitrogen | Total nitrogen | g/kg | 0.8 |
|  | Phh2o | pH Soil pH | pH | 0 |
|  | Sand | Proportion of sand particles (> 0.05 mm) in the fine earth fraction | g/100g (%) | 6.3 |
|  | Silt | Proportion of silt particles (≥ 0.002 mm and ≤ 0.05 mm) in the fine earth fraction | g/100g (%) | 0.1 |
|  | Soc | Soil organic carbon content in the fine earth fraction | g/kg | 2.1 |
|  | Ocd | Organic carbon density | kg/m³ | 0.3 |
|  | Ocs | Organic carbon stocks | kg/m² | 1.8 |


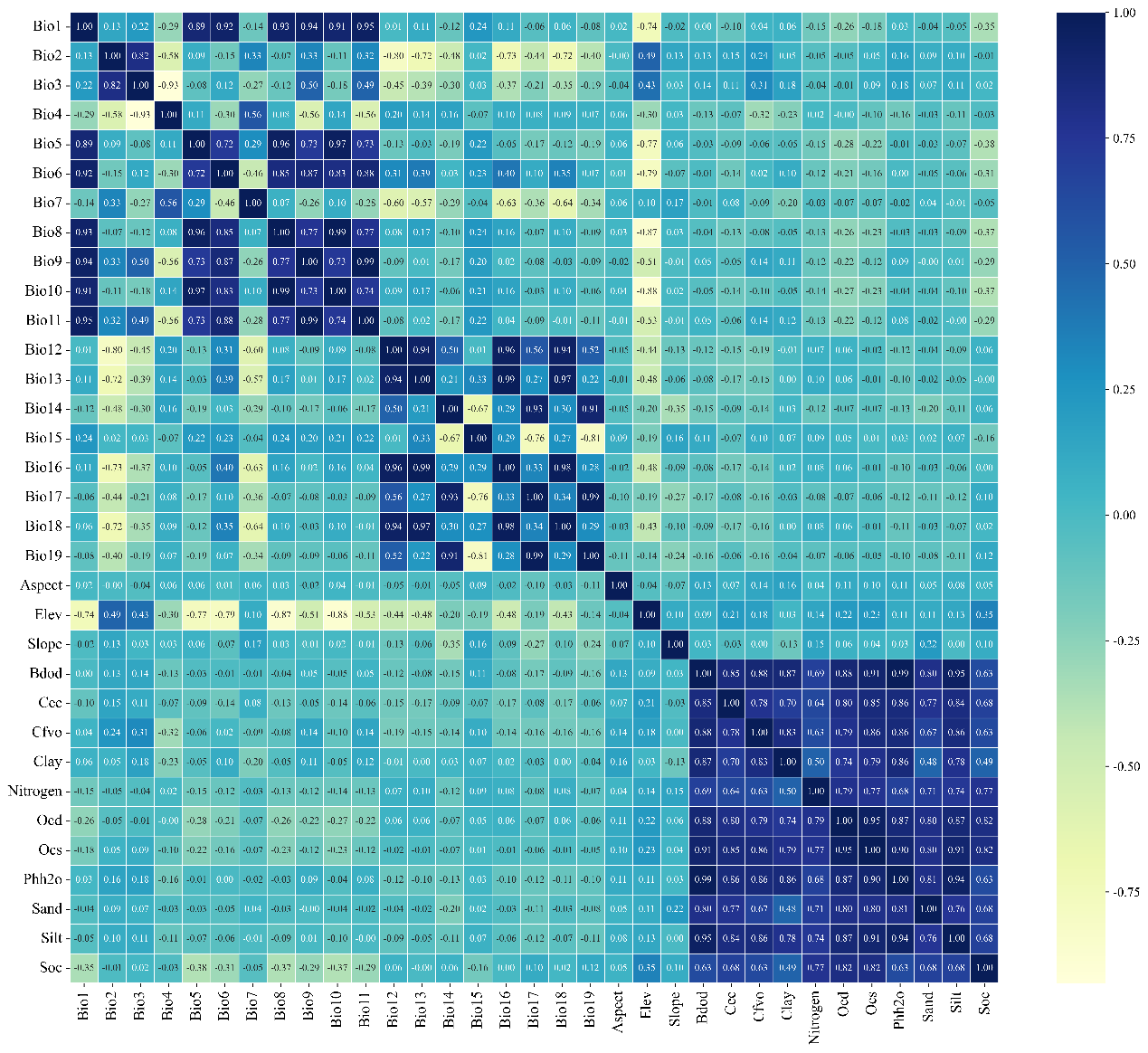


**Fig. A1.** Heatmap of 33 environment variables
